# Importance of changes in abnormal muscle responses during microvascular decompression for hemifacial spasm

**DOI:** 10.1016/j.cnp.2024.02.003

**Published:** 2024-03-08

**Authors:** Masafumi Fukuda, Yosuke Ito, Tomoyoshi Ota, Makoto Oishi

**Affiliations:** a Department of Neurosurgery, NHO Nishiniigata Chuo Hospital, Niigata-City, Niigata, Japan; b Department of Neurosurgery, Brain Research Institute, University of Niigata, Niigata-City, Niigata, Japan

**Keywords:** Abnormal muscle response, Hemifacial spasm, Mentalis muscle, Microvascular decompression, Orbicularis oculi muscle

## Abstract

**Objective:**

To determine if compression sites of the facial nerve correlate with immediate postoperative outcomes in patients with hemifacial spasm (HFS), and if changes in the waveform of abnormal muscle response (AMR) during microvascular decompression (MVD) for HFS can predict the postoperative course.

**Methods:**

In this retrospective review, we evaluated 50 patients with HFS who underwent AMR monitoring during MVD. The ratios of amplitude and duration of AMR waveforms were computed by comparing baseline with final examinations. Vascular compression sites were categorized into four portions of the facial nerve. Postoperatively, we classified patients into two groups based on symptom relief as those whose symptoms disappeared immediately (DI group), and those whose symptoms disappeared gradually (DG group).

**Results:**

The compression sites significantly correlated with postoperative outcomes at discharge (p < 0.001) but not with outcomes after 6 months of MVD. Lower duration ratios of AMRs from the mentalis muscle were significantly associated with an increased chance of classification into the DI group based on the results of multivariate logistic regression analysis (p = 0.017).

**Conclusions:**

Relationship between compression sites and immediate outcomes could provide useful information to surgeons for predicting if symptoms will resolve over long term. Moreover, changes in AMRs recorded from the mentalis muscle could predict the postoperative course of HFS.

**Significance:**

These findings can help surgeons evaluate the changes in AMR amplitude and duration during MVD for HFS.

## Highlights

• Changes in amplitude and duration of abnormal muscle response were correlated.
• Immediate outcomes following surgery were correlated with compression sites.
• Changes in duration could predict the postoperative clinical course.

## 1 Introduction

**Fig. 1:**
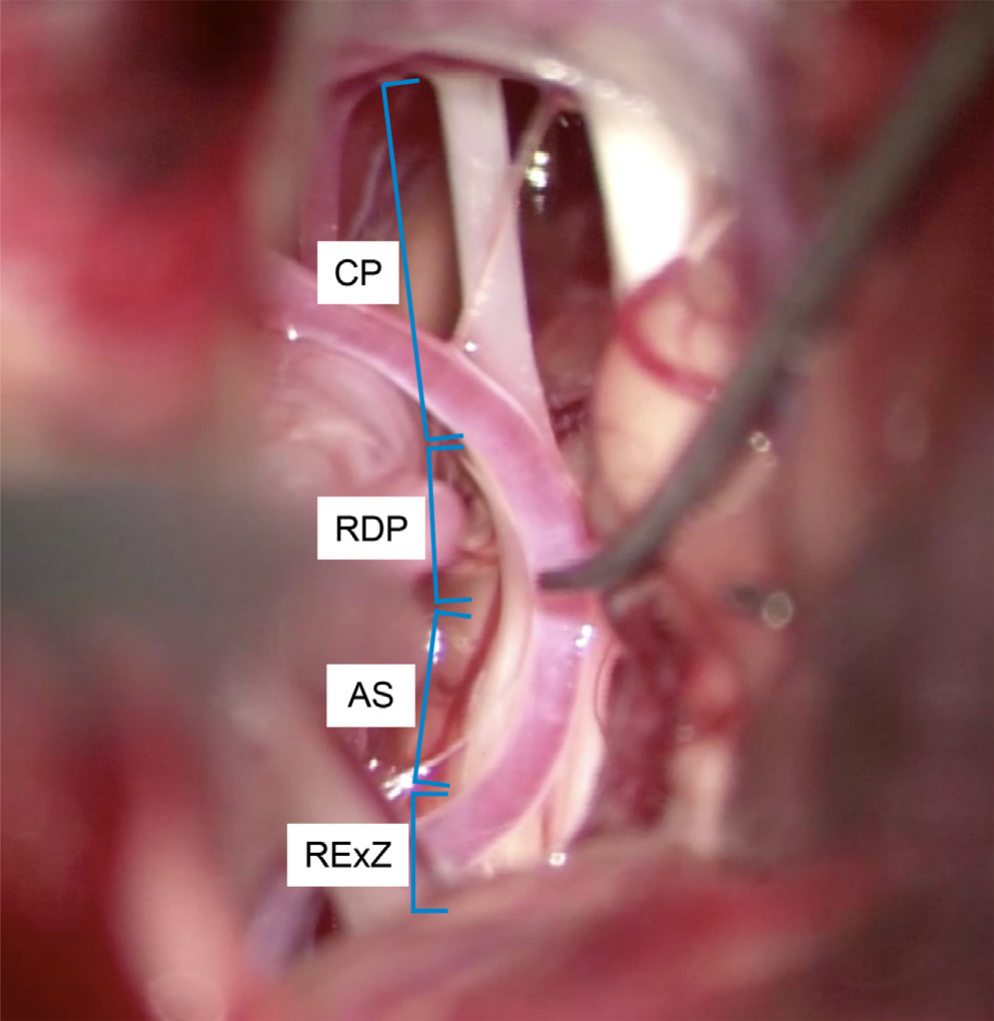
Intraoperative photograph of a patient with left hemifacial spasm (HFS). The facial nerve was anatomically categorized into the following four parts according to Campos-Benitez and Kaufmann (2008): root exit zone (RExZ), attached segment (AS), root detachment point (RDP), and cisternal portion (CP).

Hemifacial spasm (HFS) is caused by vascular compression of the facial nerve at the root exit zone (REZ), with microvascular decompression (MVD) being extensively used as an effective treatment. A comprehensive study of compression in HFS categorized the compression sites into four portions of the facial nerve: root exit point (RExP), where the facial nerve emerges from pontomedullary sulcus to the pontine surface; attached segment (AS), where the facial nerve strongly adheres to the pons surface; root detachment point (RDP), where the transition zone between central and peripheral axonal myelination exists; and cisternal portion (CP), where the compression sites are the more distal facial nerves (Fig. 1) (Campos-Benitez and Kaufmann, 2008). The term “REZ” is traditionally considered synonymous with the Obersteiner–Redlich zone and corresponds to RDP as defined by Campos-Benitez and Kaufmann (2008). They reported that 10 %, 64 %, 22 %, and 6 % vascular compression sites were at the RExP, AS, RDP, and CP portions, respectively.

Abnormal muscle response (AMR) is recorded in the facial muscles innervated by other branches of the facial nerve while stimulating a facial nerve branch in patients with HFS (Esteban and Molina-Negro, 1986, Møller and Jannetta, 1986, Møller, 1991). When the facial nerve is decompressed, AMRs either disappears or their amplitude decreases (Kim et al., 2010, Kong et al., 2007, Møller and Jannetta, 1985, Møller and Jannetta, 1986, Møller and Jannetta, 1987, Sekula et al., 2009). Therefore, intraoperative monitoring of AMR can help detect offending vessels that pressurize the facial nerve, enable effective MVD, and considerably improve postoperative results (Fukuda et al., 1997, Fukuda et al., 2010, Yamashita et al., 2005, Huang et al., 2017, Park et al., 2019). However, most studies evaluated the findings of AMR based on its presence or disappearance after facial nerve decompression (El Damaty et al., 2016, von Eckardstein et al., 2014, Joo et al., 2008, Li et al., 2012, Ying et al., 2011, Lee et al., 2017, Wei et al., 2018, Song et al., 2019). To the best of our knowledge, changes in amplitude and duration of AMRs during MVD are yet to be comprehensively analyzed.

In this study, we aimed to determine whether compression sites of the facial nerve or AMR findings correlate with postoperative outcomes at discharge and at 6 months after MVD. Additionally, regarding the postoperative period, we highlighted the outcomes based on whether the symptoms disappeared immediately (DI) or gradually only in patients who were cured at final observation. Several clinical variables including AMR findings were investigated as likely to predict the postoperative course.

## 2 Methods

### 2.1 Patients

This study was approved by the Institutional Review Board of NHO Nishiniigata Chuo Hospital (#2116) and complied with the 2013 update of the Declaration of Helsinki. This study involved 50 consecutive patients (18 men and 32 women) who underwent AMR monitoring during MVD for HFS at the NHO Nishiniigata Chuo Hospital between June 2016 and December 2022. The mean age of the patients at surgery was 56.6 (range, 31–76) years, and mean duration of symptoms was 6.2 (range, 0.5–35) years. Spasms affected the left and right sides in 32 and 18 patients, respectively. Twenty-two (44.0 %) patients had received injections of botulinum neurotoxin (BTX) before undergoing MVD. The average number of BTX injections was 7.8 (range, 1–50). MVD was performed at least 6 months after the latest BTX treatment. It was determined whether the symptoms of patients disappeared or persisted at discharge and 6 months after MVD. Additionally, the periods from surgery to disappearance of symptoms were confirmed in patients who were symptom-free. The end of the follow-up period was June 2023.

### 2.2 Surgical procedures

General neuroanesthesia was given through intravenous administration of an infusion comprising propofol and fentanyl. Muscle relaxants were not used until completion of MVD, except for intratracheal intubation. The patients were put in the lateral oblique position, with the head fixed in a neutral position and rotated slightly toward the surgeon. After dissecting the arachnoid around the glossopharyngeal and vagal nerves, the REZ of the facial nerve was ascertained using the infrafloccular approach.

The location of neurovascular compression was classified as RExP, AS, RDP, or CP according to the criteria defined by Campos-Benitez and Kaufmann (2008). When the offending vessels stuck into the pontomedullary sulcus, the compression sites were inferred as RExP. The AS, RDP, and CP compression sites were identified by visually confirming an indentation of the facial nerve. The compression sites could be easily identified in patients with a single culprit vessel, even if the facial nerve did not have a clear indentation. However, in some patients with multiple culprit vessels, real-time intraoperative changes in AMRs, in the culprit vessels compressing the facial nerve, helped in identifying the true compression sites.

We shifted offending vessel off the facial nerve using its adhesion to the dura covering the pyramidal bone or inserting a small piece of shredded Teflon felt between the vessels and brainstem or flocculus. In a few patients, especially those with distal compression sites (RDP or CP), the Teflon felt were inserted between the vessels and facial nerve.

### 2.3 Intraoperative monitoring

Neuromaster (Nihon Kohden Corp., Tokyo, Japan) was used to perform intraoperative monitoring. Brainstem auditory evoked potentials were monitored in all patients until dural closure to preserve hearing and a <50 % reduction in V waves was used as a warning sign.

Subdermally inserted paired stainless steel needle electrodes at the zygomatic arch and lower edge of the mandibular bone were used as stimulation electrodes of the zygomatic and mandibular branches, respectively. The other paired stainless steel needle electrodes were subdermally inserted into the orbicularis oculi and mentalis muscles as recording electrodes. AMRs were recorded from the orbicularis oculi and mentalis muscles by stimulating the mandibular and zygomatic branches, respectively. AMRs were recorded using amplifiers with a frequency band of 5–3000 Hz.

Typically, the zygomatic branch was stimulated, and waves from the mentalis muscle were recorded for AMR monitoring (Lee et al., 2018). Only a few studies have reported AMRs recorded from the orbicularis oculi muscle by stimulating the mandibular branch (Fukuda et al., 1997, Fukuda et al., 2010, Yamashita et al., 2005, Nakayama et al., 2021). AMRs from the mentalis muscle have been frequently used for intraoperative monitoring to obtain more stable responses than those obtained from other muscles (Miao et al., 2020). We used two muscles for recording AMR under MVD for HFS because we could not record AMRs from the mentalis muscles during the surgery in some of the patients with HFS. AMRs from the multibranch facial nerve stimulation were more frequently detected than those from single-branch stimulation; consequently, the methods were useful in confirming MVD completion intraoperatively (Miao et al., 2020).

AMR recording was obtained upon opening the dura, retracting the cerebellum, operating and transposing the offending vessels, and terminating the microsurgical procedure and dura closure. Upon operating the culprit vessels, AMRs were frequently recorded at approximately 1 Hz. In some cases, real-time changes in the duration and amplitude of AMRs were noted intraoperatively. These findings helped the surgeons confirm that the culprit vessels caused HFS. If the AMRs completely disappeared or waveform had changed compared with the baseline, MVD completion was confirmed. Patients in whom AMR disappeared after draining the cerebrospinal fluid or while placing retractors in the cerebellum were continuously monitored for AMR at the same intensity that was used during stimulation until dura closure. If the AMR did not deviate from the baseline response despite decompression, we explored other regions to determine other possible offending vessels to achieve completion of facial nerve decompression. In cases where the other culprit vessels could not be identified despite additional investigations, we did not further manipulate even if there were changes in AMRs from the mentalis or orbicularis oculi muscle.

### 2.4 Evaluation of surgical outcomes and AMRs

In this study, patients who were symptom-free at the final follow-up were categorized into two groups based on the time taken for the disappearance of symptoms, i.e., immediately after surgery (DI group) or gradual improvement until complete resolution (DG group).

**Fig. 2:**
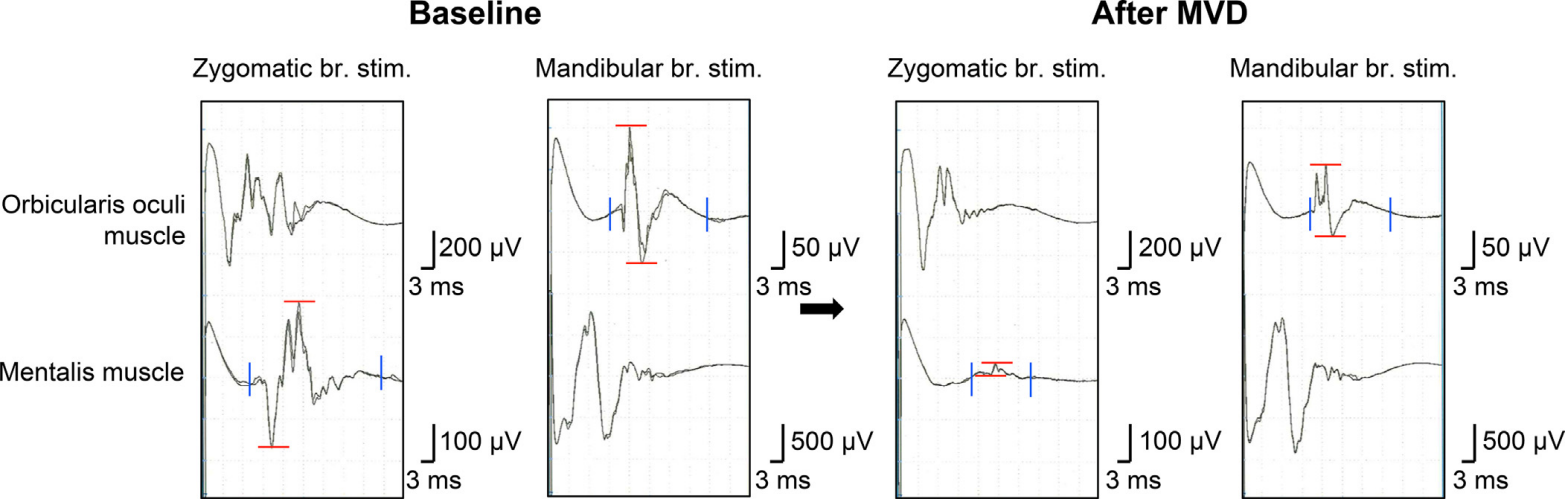
Measurements of the amplitude and duration of abnormal muscle responses (AMRs). The changes in AMRs obtained from the orbicularis oculi and mentalis muscles by stimulating the mandibular branch and zygomatic branch of the facial nerve at the baseline (left) and after microvascular decompression (MVD, right). Two horizontal lines in each wave show minimum and maximum amplitudes and the other two vertical lines show the onset and terminal duration. Both duration and amplitude in AMRs recorded from the orbicularis oculi and mentalis muscles decreased after MVD as compared with those at baseline. In AMRs obtained from the orbicularis oculi muscle and mentalis muscle, the duration ratios were 59.2% and 33.7% and amplitude ratios were 52.5% and 9.2%, respectively.

AMR waveforms recorded at baseline and dural closure from the orbicularis oculi and mentalis muscles were compared. Amplitude was defined as the range from minimal to maximal values of the AMR waveform, and duration was defined as the range from onset to terminal values (Fig. 2). The onset value of AMR was measured as stimulation artifact and current spread were not included in the AMR waveform and the terminal value was measured as the waveform recovered to the baseline. These values were automatically measured by Neuromaster (Nihon Kohden Corp., Tokyo, Japan). Amplitude and duration ratios were calculated using the following formula:

$$\begin{array}{c} \text{Amplitude}\;\text{ratio}\left(\%\right)=\left(\text{final}\;\text{maximum}-\text{minimum}\;\text{values}\right)/(\text{baseline}\;\text{maximum}-\text{minimum}\\ \text{values})\times 100 \end{array}$$

$$\text{Duration}\;\text{ratio}\left(\%\right)=\left(\text{final}\;\text{terminal}-\text{onset}\;\text{values}\right)/\left(\text{baseline}\;\text{terminal}-\text{onset}\;\text{values}\right)\times 100$$

### 2.5 Statistical analysis

IBM SPSS, Statistics version 29 (IBM Corp., Armonk, NY, USA) was used for statistical analyses. The chi-squared test was used to evaluate whether the outcomes at discharge or at 6 months after surgery were correlated with the compression sites, and unpaired *t*-test was used to evaluate whether the outcomes were correlated with AMR findings. We did not further investigate if there were changes in AMRs from the mentalis or orbicularis oculi muscle. Patient’s age, duration of history, BTX treatment history (yes vs. no), relation of the vertebral artery (VA) to compression (yes vs. no), amplitude ratios of AMRs obtained from the orbicularis oculi and mentalis muscles, and duration ratios of AMRs obtained from the orbicularis oculi and mentalis muscles were tested as independent factors postoperatively (DI vs. DG) using univariate and multivariate logistic regression analyses. The chi-squared and Fisher exact tests or Mann–Whitney *U* test were used to compare the factors between groups. A p value of <0.05 was considered statistically significant.

## 3 Results

### 3.1 Surgical results

RExP, AS, RDP, and CP were the compression sites in 18 (36.0 %), 19 (38.0 %), 9 (18.0 %), and 4 (8.0 %) patients, respectively.

In 46 of the 50 (92.0 %) patients, HFS completely disappeared at final observation. Other two patients experienced recurrence of symptoms after 1 and 5 months of surgery and received BTX treatment. The remaining two patients experienced a reduction in symptoms at 10 % and 20 % intensity compared with that before surgery, but HFS persisted until 63 and 23 months after MVD. They were satisfied with their current conditions and received no additional treatments for residual symptoms of HFS. Although three patients had transient facial palsy, two had hearing disturbance, and another two had mild swallowing disturbance, no major postoperative complications were observed in any of the patients.

**Fig. 3:**
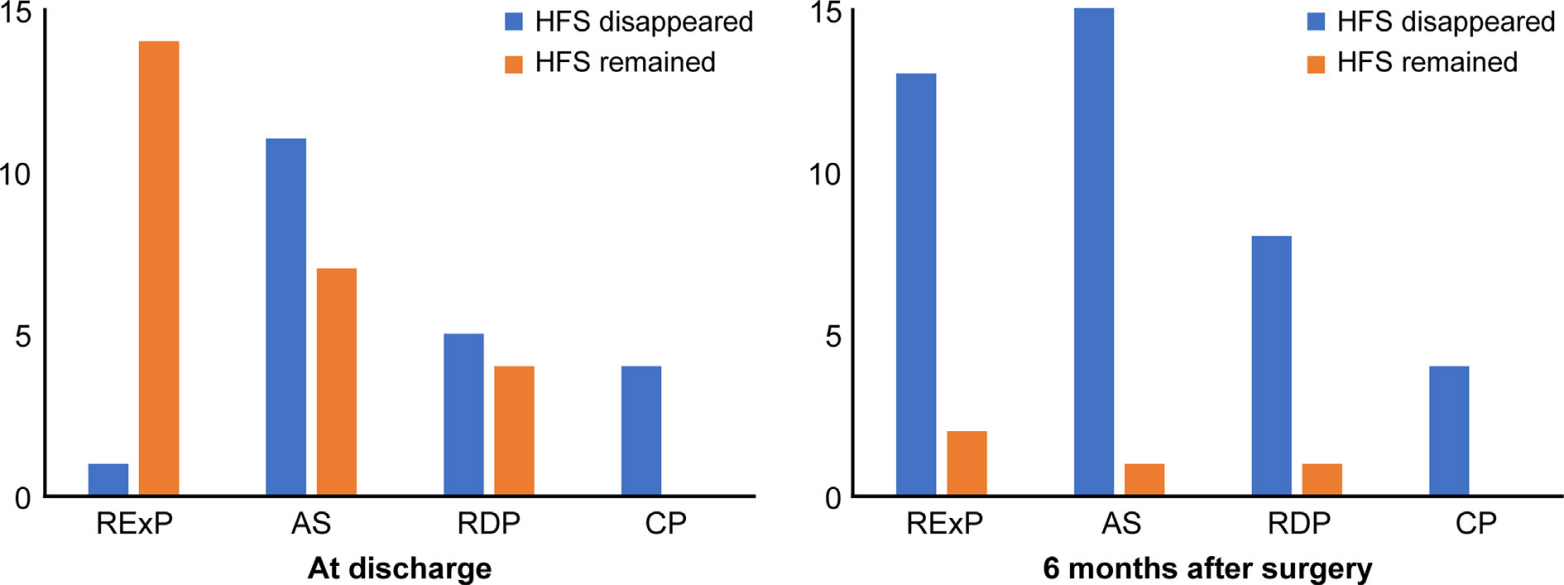
Relationship between the compression sites and clinical outcomes at discharge and after 6 months of surgery (n = 46). The compression sites showed significant correlation with clinical outcomes at discharge (p = 0.001). The number of patients with remained symptoms decreased from the RExP to CP (left); however, they did not correlate with clinical outcomes after 6 months of surgery (p = 0.530, right).

In 46 patients whose symptoms were resolved at final observation, 21 (45.7 %) experienced no symptoms at discharge and 42 (91.3 %) had no symptoms after 6 months of surgery (Table 1). The compression sites were significantly correlated with postoperative outcomes at discharge (Fig. 3, p = 0.001), whereas they did not correlate with postoperative outcomes after 6 months of surgery (p = 0.530) (Fig. 3).

**Table 1:** Relationship between AMR findings and clinical outcomes at discharge and after 6 months of surgery (n = 46).

Compression sites and AMR findings	At discharge		P value	6 months after surgery		P value
	HFS disappeared	HFS remained		HFS disappeared	HFS remained	
n (%)	21 (45.7)	25 (54.3)		42 (91.3)	4 (8.7)	
Duration ratio of AMR from the orbicularis oculi muscle. in % (±SD)	13.4 ± 27.8 (n = 19)	28.5 ± 39.1	0.008	21.2 ± 34.2 (n = 40)	29.8 ± 48.8	0.480
Amplitude ratio of AMR from the orbicularis oculi muscle. in % (±SD)	3.2 ± 8.0 (n = 19)	32.5 ± 55.5	<0.001	16.2 ± 34.0 (n = 40)	56.6 ± 105.7	<0.001
Duration ratio of AMR from the mentalis muscle. in % (±SD)	7.3 ± 15.3	47.1 ± 31.9	<0.001	26.4 ± 32.3	55.6 ± 21.3	0.262
Amplitude ratio of AMR from the mentalis muscle. in % (±SD)	7.7 ± 22.2	42.9 ± 45.1	0.002	25.1 ± 41.2	45.4 ± 24.3	0.501

Abbreviations: AMR, abnormal muscle response; HFS, hemifacial spasm; RExP, root exit point; AS, attached segment; RDP, root detachment point; CP, cisternal portion.

Of the 46 patients in whom HFS completely disappeared at final observation, 17 (37.0 %) and 29 (63.0 %) were assigned to the DI and DG groups, respectively. In the DG group, symptoms of patients resolved completely within 0.2–9.0 (mean, 3.1) months of MVD. In 4 out of 29 patients in DG group, HFS did not yet disappear when examined at 6 months after surgery. Three out of four patients finally experienced symptom-free 8 months after surgery, and the remaining one patient experienced symptom-free 9 months after surgery.

### 3.2 AMR findings

AMRs were obtained from the mentalis muscle in all patients; however, AMRs were not recorded from the orbicularis oculi muscle in three patients. These three patients had a history of receiving BTX injections for 2, 10, and 35 times before surgery.

Analysis of the relationship between AMR findings and preoperative BTX treatment (Nakayama et al., 2021) revealed that AMRs from the orbicularis oculi muscle were not obtained in more patients in the BTX group than in non-BTX group. Additionally, the AMR recordings from the orbicularis oculi muscles were poorer in patients who received more than four BTX injections than in those who received less than four BTX injections. Moreover, two of the three patients in our series had received BTX injections for more than four times before surgery.

**Fig. 4:**
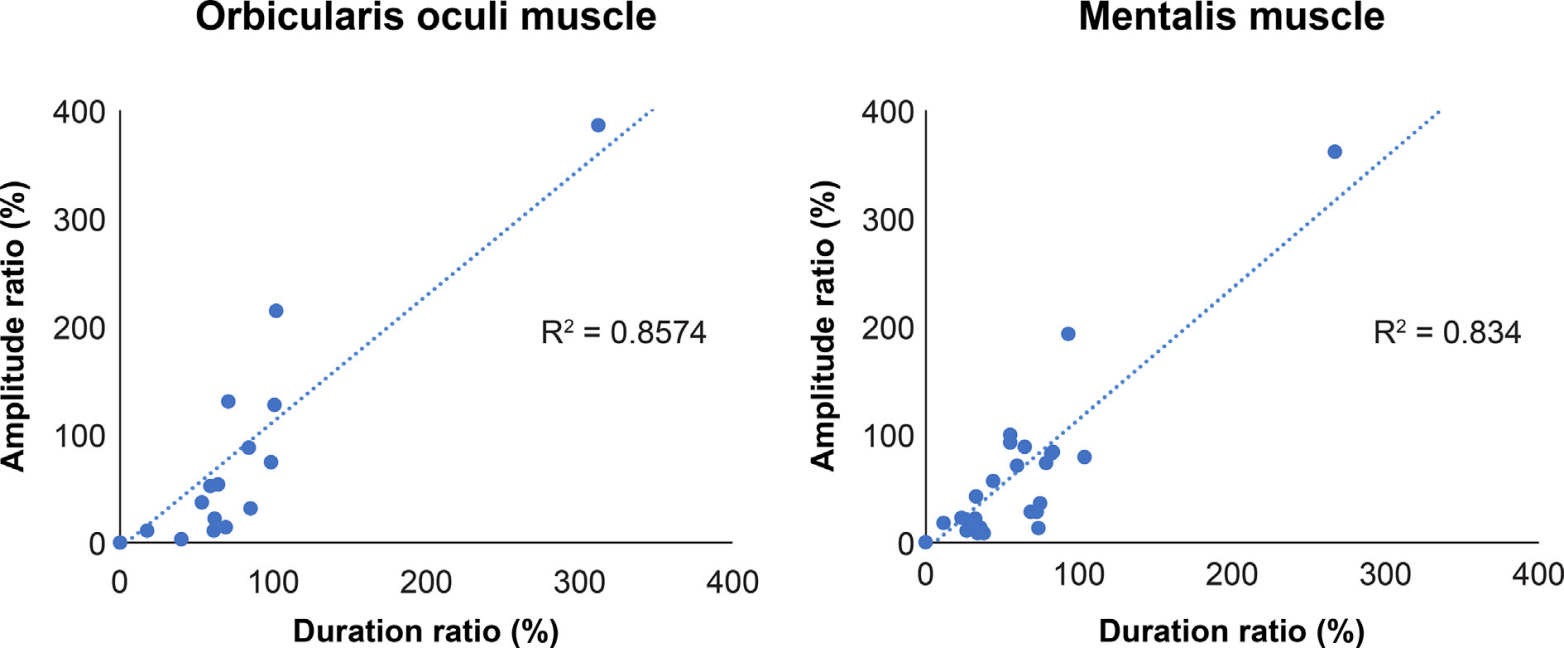
Scatterplot of the correlation between the amplitude and duration ratios of AMRs recorded from the orbicularis oculi (left) and mentalis muscle (right). In both muscles, the two ratios showed strong positive correlations (p < 0.001).

In both muscles, amplitude ratios were strongly correlated with duration ratios (orbicularis oculi muscle: n = 47, r = 0.926, p < 0.001; mentalis muscle: n = 50, r = 0.913, p < 0.001; Fig. 4).

Table 1 presents the AMR findings in each group with or without HFS symptoms at discharge and at 6 months after surgery. All variables were significantly correlated with outcomes at discharge, whereas those variables except for amplitude ratios that were obtained from the orbicularis oculi muscle did not correlate with those after 6 months of surgery (Table 1).

### 3.3 Postoperative outcomes

The final analysis included 46 patients, with a mean age and disease duration of 56.5 (standard deviation [SD]: 10.8) and 6.3 (SD: 6.9) years, respectively at the time of surgery. Furthermore, 14 (30.4 %) patients had received BTX 6 months before surgery and 14 (30.4 %) had the VA involved in compression (Table 2). The means (SD) of the duration and amplitude ratios of the orbicularis oculi muscle were 22.0 % (35.1) and 19.9 % (44.3), respectively, whereas those of the mentalis muscle were 28.9 % (32.4) and 26.9 % (40.2), respectively (Table 2). Patients in the DI group (n = 11, 64.7 %) underwent BTX treatments more frequently than those in DG group (n = 8, 27.6 %) (p = 0.028).

**Table 2:** Characteristics of patients whose symptoms immediately and gradually disappeared.

Characteristics	Total	Disappeared immediately (DI)	Disappeared gradually (DG)	PValue*
No. of patients (%)	46 (100)	17 (37.0)	29 (63.0)	
Mean age at op, in years (±SD)	56.5 ± 10.8	57.1 ± 11.0	55.4 ± 11.2	0.618
Mean disease duration, in years (±SD)	6.3 ± 6.9	8.4 ± 9.2	5.0 ± 4.8	0.167
No. of patients with BTX (%)	19 (41.3)	11 (64.7)	8 (27.6)	0.028
No. of patients in whom VA was related to compression (%)	14 (30.4)	4 (23.5)	10 (34.5)	0.520
Duration ratio of AMR from the orbicularis oculi muscle. in % (±SD)	22.0 ± 35.1 (n = 44)	17.0 ± 30.4 (n = 15)	24.6 ± 37.5	0.476
Amplitude ratio of AMR from the orbicularis oculi muscle. in % (±SD)	19.9 ± 44.3 (n = 44)	4.1 ± 8.9 (n = 15)	28.0 ± 52.7	0.023
Duration ratio of AMR from the mentalis muscle. in % (±SD)	28.9 ± 32.4	9.1 ± 16.6	40.6 ± 33.8	<0.001
Amplitude ratio of AMR from the mentalis muscle. in % (±SD)	26.9 ± 40.2	9.6 ± 24.4	37.0 ± 44.4	0.010

Abbreviations: AMR, abnormal muscle response; BTX, botulinum neurotoxin; VA, vertebral artery.

* Significance set at p < 0.05 in the univariate analysis performed using the chi-square and Fisher exact tests and the Mann–Whitney *U* test; see Statistical Analysis.

AMR findings recorded from the orbicularis oculi muscle were unavailable for 2 of the 17 patients in the DI group. Therefore, analysis of the postoperative course and AMR findings obtained from the orbicularis oculi muscle was performed for 15 and 29 patients in the DI and DG groups, respectively. Although the duration ratios were not significantly different between the groups, the amplitude ratios in the DI group were significantly lower than those in DG group (mean ± SD: DI group, 4.1 ± 8.9, DG group, 28.0 ± 52.7; p = 0.023; Table 2). Analysis of the postoperative course and AMR findings recorded from the mentalis muscle showed that both duration and amplitude ratios (mean ± SD) in the DI group were significantly lower than those in DG group (duration ratios in DI group: 9.1 ± 16.6; duration ratios in DG group, 40.6 ± 33.8; p < 0.001; amplitude ratios in DI group: 9.6 ± 24.4; amplitude ratios in DG group, 37.0 ± 44.4; p = 0.010; Table 2).

Because strong correlations were observed between the amplitude and duration ratios of AMRs obtained from the orbicularis oculi and mentalis muscles, both amplitude and duration ratios of AMRs were used as independent variables for multivariate analysis. The multivariate logistic regression analysis did not show an association between patient characteristics (age at surgery, duration, BTX administration, VA involvement, and amplitude ratios of AMR) and postoperative outcomes of patients with HFS who underwent MVD. The duration ratio of the mentalis muscle was associated with postoperative outcome in patients with HFS who underwent MVD (Table 3). All other variables were not associated with the postoperative outcomes in patients with HFS who underwent MVD.

**Table 3:** Multivariate logistic regression illustrating the association between patient characteristics (including duration ratios) and postoperative outcomes among patients with hemifacial spasm who underwent microvascular decompression, 2016–2022.

Variables	Odds ratio	95 % CI for odds ratio		P Value*
		Lower	Upper	
Age at op	0.989	0.928	1.054	0.738
Duration	0.948	0.823	1.091	0.453
BTX treatment	0.459	0.099	2.130	0.320
VA compression	1.399	0.261	7.496	0.695
Duration of AMR from the orbicularis oculi muscle	0.797	0.069	9.223	0.856
Duration of AMR from the mentalis muscle	101.926	2.325	4467.683	0.017

Abbreviations: BTX, botulinum neurotoxin; VA, vertebral artery; AMR, abnormal muscle response.

n = 44.

* Significance set at p < 0.05 on the multivariate logistic regression analysis performed using the log-likelihood ratio test.

## 4 Discussion

This study aimed to assess if compression sites of the facial nerve were correlated with postoperative outcomes at discharge and at 6 months after MVD in patients with HFS. Additionally, the study evaluated postoperative outcomes of patients with HFS based on whether the symptoms disappeared immediately or gradually in patients who were considered cured at the final observation. Regarding the AMR findings, we did not evaluate if they disappeared or persisted but noticed changes in the amplitude and duration of the AMR waveform after MVD. Based on our results, the compression sites and AMR findings were significantly correlated with postoperative outcomes only at discharge, and only the duration ratios of AMRs from the mentalis muscle were related to postoperative outcomes.

### 4.1 Changes in amplitude and duration of AMRs

Most studies have employed “disappeared or remained” as intraoperative AMR findings to evaluate the usefulness of AMR monitoring for confirming the completion of MVD (El Damaty et al., 2016, von Eckardstein et al., 2014, Joo et al., 2008, Li et al., 2012, Ying et al., 2011, Lee et al., 2017, Wei et al., 2018, Song et al., 2019). To the best of our knowledge, this is the first study where both the amplitude and duration of AMRs are examined and compared at baseline and after decompression of the facial nerve. In this study, strong correlations were found between the amplitude and duration ratios of AMRs from the orbicularis oculi and mentalis muscles. The results suggested that the duration and amplitude could be shortened by relieving facial nerve compression. In patients with HFS, AMRs with longer duration waveforms indicated a higher degree of demyelination and excitability of the facial nucleus as longer AMR duration likely includes the F waves of the facial muscles (Ishikawa et al., 1996a, Ishikawa et al., 1996b). The intraoperative decrease in the duration and amplitude of AMRs after MVD requires further confirmation.

### 4.2 Postoperative outcomes and compression sites

In a previous study of compression sites (Campos-Benitez and Kaufmann, 2008), RExP was 10 % and AS was 64 %, and in the current study, RExP was 36 % and AS was 38 %. Herein, we inferred the compression sites as RExP if the offending vessels stuck into the pontomedullary sulcus because the indentation at RExP could not be confirmed in some patients. Additionally, it can often be difficult to identify if the offending vessels compressed at the RExP or AS due to the border was not obvious. These reasons are attributed to the rates of compression sites being different between the results from previous and current study.

As per the results, the compression sites of the facial nerve were correlated with postoperative outcomes at discharge. The findings suggest that patients with compressed RExZ are less frequent to become symptom-free immediately after MVD, compared with those with compressed distal sites of the facial nerve, such as RDP or CP. Considering the distance between the facial motor nucleus existing within the pons and compression sites, the results indicating that being near to the facial nucleus correlated with persistence of symptoms in the early phase after surgery could support the findings that compression sites closer to the RExP were likely to be more closely related to hyperexcitability of the facial nucleus. In an animal study using rats (Møller and Sen, 1990), chronic electrical stimulation of the facial nerve near the brainstem induced an AMR, which was similar to what is observed in patients with HFS. Recording from the facial motor nucleus in the rats showed the physiological abnormalities, which were produced due to chronic antidromic neural activity resulting from compression near the brainstem. The lack of correlation between the compression sites and postoperative outcomes after 6 months of MVD in this study could support that the hyperexcitability of facial nucleus was likely to be normalized in the long term, even in patients whose compression sites were RExP.

### 4.3 Role of AMR monitoring during MVD

Although numerous studies have explored if intraoperative AMR findings can predict postoperative outcomes in patients with HFS, their conclusions are controversial (El Damaty et al., 2016, Joo et al., 2008, Li et al., 2012). Recently, a *meta*-analysis of the utility of intraoperative AMR monitoring in MVD showed that persistent AMR is associated with a high rate of immediate and long-term persistence of facial spasm. Therefore, the adequacy of decompression should be thoroughly investigated before closing in cases where intraoperative AMR persists (Thirumala et al., 2020). Conversely, the other *meta*-analysis study about the prognostic value of AMR during MVD revealed that AMR disappearance demonstrated limited prognostic value for better short-term outcomes but is ineffective in predicting long-term outcomes (Zhang et al., 2020). The HFS outcomes following MVD are exclusively attributed to the surgical skill of separating the offending vessels at an appropriate distance from the compression sites of the facial nerve regardless of the AMR findings. Most studies on correlations between AMR findings and long-term outcomes have reported the recurrence of symptoms because of recompression of the offending vessels owing to inadequate decompression (El Damaty et al., 2016, Li et al., 2012, Lee et al., 2017, Wei et al., 2018, Song et al., 2019). AMR monitoring is useful for identifying the offending vessels and confirming intraoperative completion of MVD (Kong et al., 2007, Lee et al., 2017). We used stimulation rates of 1 Hz during the manipulation of culprit vessels. If the culprit vessels were truly the offending vessels, the AMR waveforms changed the amplitude and latency in real-time. Observing the instability of AMR waveforms can probably provide surgeons with important information to identify the offending vessels intraoperatively (Sun et al., 2022).

### 4.4 AMR changes and postoperative course

Some studies have demonstrated that gradual postoperative improvement in HFS is correlated with preoperative duration of symptoms (Hyun et al., 2010, Shin et al., 1997, Xu et al., 2020). Longer compression time can increase the time of regeneration of facial nerve demyelination or normalization of the excitability of the facial nucleus. In this study, the duration of history was not significantly correlated with the postoperative clinical course; however, HFS was immediately cured in patients who received BTX treatment before surgery. The disparity between the results of previous and current study may be attributed to the sample size and effects of previous BTX injections on facial muscles that facilitate an immediate relief of muscle twitching, as these effects persisted even when the surgery was performed after at least 6 months of receiving the last BTX injection (Nakayama et al., 2021).

Previous studies have reported that patients with persistent AMRs after MVD tended to experience delayed HFS resolution (Kong et al., 2007, Xu et al., 2020, Hatem et al., 2001, Kiya et al., 2001). In this study, we not only investigated the amplitude but also the duration of AMRs in each patient and found that long duration of AMRs from the mentalis muscle after MVD was strongly correlated with the interval between the surgery and complete relief of symptoms. Although AMRs may occur mainly because of facial nerve demyelination at the vascular compression sites, the hyperexcitability of the facial nucleus might be related to the origin of AMRs to some extent, specifically in patients in whom AMRs did not disappear, and there was only a small reduction in the duration of AMRs despite MVD completion intraoperatively. The results reveal that the distances from the pontomedullary sulcus to compression sites are significantly correlated with immediate outcomes after MVD. Additionally, these results support the findings that compression sites closer to the pons increase excitability of the facial nucleus and interval until normalization of the facial motor neurons. We must focus on the “disappearance or persistence” of AMRs and waveform changes, including the amplitude and duration of AMRs during MVD. The duration of AMRs obtained from the mentalis muscle is likely useful in predicting the postoperative course based on whether the HFS symptoms would disappear immediately or gradually.

This study has some limitations. First, it is a retrospective study, and the sample size is relatively small. Second, the confirmation of the compression sites was probably not objective and could have been biased. However, the compression sites were intraoperatively identified as per the criteria developed by Campos-Benitez and Kaufmann (2008) in all patients with HFS. We certainly think that the distance from the pons to the compression sites was likely to relate to the immediate outcomes in our results. Third, the rates of number of patients in the DG group were much higher than those reported in previous studies. The delayed rate of cure was 63 % in our study, whereas it was 10.5 %–37.4 % in previous reports (Lee et al., 2017, Shin et al., 1997, Xu et al., 2020). This disparity may be attributed to the fact that we inferred slight spasms only within the lower eyelid after MVD as residual symptoms and classified them into the DG group. Further studies with larger sample sizes are warranted to confirm the present results.

## 5 Conclusions

In AMR monitoring during MVD for HFS, waveform changes in amplitude and duration should be evaluated. Moreover, the relationship between immediate outcomes after MVD and compression sites could likely provide useful information to surgeons confirming whether the operation is successful or not. The changes in the duration of AMRs recorded from the mentalis muscle could predict the postoperative course based on whether the symptoms disappeared immediately or gradually. This is probably useful in explaining postoperative outcomes in patients with HFS.

## CRediT authorship contribution statement

**Masafumi Fukuda:** Conceptualization, Methodology, Project administration, Writing draft and review. **Yosuke Ito:** Resources, Data curation. **Tomoyoshi Ota:** Resources, Data curation. **Makoto Oishi:** Supervision.

## Ethical approval and informed consent

This study was approved by the Institutional Review Board of NHO Nishiniigata Chuo Hospital (#2116). All patients and their families were informed about intraoperative monitoring and obtained informed consent on the previous day of surgery.

## Funding

This research did not receive any specific grant from funding agencies in the public, commercial, or not-for-profit sectors.

## Author contributions

M Fukuda conceived and designed the study, collected and contributed data, performed the analysis, and wrote the paper. Y Ito, T Ota, and M Oishi assisted with data collection. All authors have approved the final article.

## Declaration of interest

None.

## Data availability

The datasets used and/or analyzed during the current study are available from the corresponding author on reasonable request.
